# Advances in Sodium Channel Modulation in Epilepsy Therapy: Focus on Eslicarbazepine, Lacosamide and Cenobamate

**DOI:** 10.3390/biomedicines14081694

**Published:** 2026-07-28

**Authors:** Monika Rudkowska, Jarosław Mołdoch, Olga Wronikowska-Denysiuk, Monika Agacka-Mołdoch, Anna Pradiuch, Karolina Wojtunik-Kulesza

**Affiliations:** 1 Independent Laboratory of Behavioral Studies, Medical University of Lublin, 1 Chodźki, 20-400 Lublin, Poland; olga.wronikowska-denysiuk@umlub.edu.pl; 2 Department of Phytochemistry, Institute of Soil Science and Plant Cultivation, State Research Institute, ul. Czartoryskich 8, 24-100 Pulawy, Poland; jmoldoch@iung.pulawy.pl; 3 Department of Biotechnology and Plant Breeding, Institute of Soil Science and Plant Cultivation, State Research Institute, ul. Czartoryskich 8, 24-100 Pulawy, Poland; magacka@iung.pulawy.pl; 4 Department of Pathophysiology, Medical University of Lublin, ul. Jaczewskiego 8b, 20-090 Lublin, Poland; anna.pradiuch@umlub.edu.pl; 5 Department of Inorganic Chemistry, Medical University of Lublin, Chodźki 4A, 20-093 Lublin, Poland; karolina.wojtunik-kulesza@umlub.edu.pl

**Keywords:** epilepsy, antiseizure medications, voltage-gated sodium channels, eslicarbazepine acetate, lacosamide, cenobamate, focal seizures

## Abstract

**Background/Objectives:** Voltage-gated sodium channels (VGSCs) are among the most important molecular targets in epilepsy therapy. Unlike classical antiseizure medications (ASMs), newer sodium channel modulators selectively affect slow inactivation or persistent sodium currents, potentially improving seizure control while preserving physiological neuronal activity. This review summarizes the pharmacology, mechanisms of action, clinical efficacy, and therapeutic potential of eslicarbazepine acetate, lacosamide, and cenobamate. **Methods:** A narrative review of published clinical trials, meta-analyses, and real-world evidence was conducted. The analysis focused on sodium channel modulation, pharmacokinetic properties, efficacy in monotherapy and adjunctive therapy, and safety profiles in focal epilepsy. **Main findings of the review:** Eslicarbazepine acetate and lacosamide primarily enhance slow inactivation of VGSCs, suppressing pathological repetitive neuronal firing with limited effects on normal neuronal signaling. Both agents demonstrated efficacy in monotherapy and add-on therapy, with favorable pharmacokinetic properties and a relatively low potential for drug–drug interactions. Cenobamate represents a novel therapeutic approach through preferential inhibition of persistent sodium currents combined with positive allosteric modulation of GABA_A_ receptors. Clinical trials and real-world studies demonstrated high responder and seizure freedom rates, particularly in patients with drug-resistant focal epilepsy. The most common adverse effects across these agents included dizziness, somnolence, fatigue, and gastrointestinal symptoms, while notable safety concerns included hyponatremia with eslicarbazepine acetate and drug interactions or dose-dependent adverse effects with cenobamate. **Conclusions:** Recent advances in sodium channel modulation have expanded therapeutic options for focal epilepsy and support the development of more selective, mechanism-based ASM therapies. Eslicarbazepine acetate, lacosamide, and cenobamate demonstrate favorable efficacy and tolerability profiles and may improve seizure control in patients with drug-resistant epilepsy.

## 1. Introduction

Epilepsy is one of the most common chronic neurological disorders worldwide, affecting approximately 50 million people and representing a major global public health challenge [1,2]. The disorder is characterized by the occurrence of recurrent, unprovoked seizures. These seizures are the clinical manifestation of abnormal, excessive, and synchronous electrical activity of neurons. The neurochemical and physiological basis of epileptic seizures is rooted in a disruption of the balance between two fundamental processes in the central nervous system: excitation and inhibition. Two neurotransmitters play a central role in this homeostasis: the excitatory glutamate and the inhibitory gamma-aminobutyric acid (GABA) [3].

Modern pharmacotherapy for epilepsy is built upon systematic drug trials, the primary objective of which is to achieve complete seizure freedom while minimizing adverse effects. Monotherapy serves as the established standard of care for patients with newly diagnosed epilepsy, enabling approximately 47% to 50% of individuals to attain seizure-free status with the very first anti-seizure medication (ASM). The selection of a specific agent for monotherapy must be highly individualized, based on a precise diagnosis of seizure type and epilepsy syndrome while accounting for patient-specific factors such as age, gender, and comorbidities. Key advantages associated with the use of a single drug include superior treatment tolerability, lower toxicity, an absence of pharmacological interactions, and improved patient adherence to the therapeutic regimen. In clinical scenarios where the initial trial is ineffective or poorly tolerated, the introduction of a second sequential monotherapy is recommended, which provides remission for an additional 13% to 20% of the patient population [4,5].

Despite these efforts, nearly one-third of patients do not achieve sustained seizure control after two appropriately selected and tolerated monotherapy trials, leading to a diagnosis of drug-resistant epilepsy (DRE). In these cases, the implementation of polytherapy is required, guided by the concept of “rational polytherapy”. This strategy involves the combination of medications with complementary, rather than overlapping, mechanisms of action to achieve therapeutic synergy, thereby increasing efficacy without a proportional increase in cumulative toxicity [5,6,7].

Although the introduction of newer-generation ASMs has significantly improved the safety profile of combination therapy through their linear pharmacokinetics and reduced potential for drug–drug interactions, polytherapy continues to carry inherent risks [4,5]. The most significant concerns include cumulative adverse effects, which may encompass cognitive impairment, behavioral changes, and negative impacts on mood and psychological functioning. Furthermore, polytherapy complicates the dosing schedule, which can lead to reduced adherence and increased healthcare costs [4,7]. The ultimate determination of whether to continue with additional polytherapy trials or to transition toward non-pharmacological methods, such as epilepsy surgery or neurostimulation, must always prioritize the patient’s comprehensive clinical profile and overall quality of life [6,7].

The activity of voltage-gated sodium channels (VGSCs) is crucial for the generation and propagation of action potentials in the nervous system. In the context of epilepsy, dysfunctions of these channels (known as channelopathies) are recognized as a significant pathophysiological factor contributing to neuronal hyperexcitability and hypersynchronization [8]. VGSC dysfunction plays a key role in both excitatory neurons and inhibitory circuits. In excitatory neurons, abnormal function enhances postsynaptic potentials and increases the capacity for repetitive firing, promoting burst firing. In inhibitory interneurons, mutations in sodium channels (e.g., Nav1.1) can lead to a loss of function, selectively reducing sodium current in these neurons, which weakens GABAergic inhibition and thereby increases the excitability of the entire neuronal circuit [9]. Neurons exhibit two main types of sodium currents: the transient sodium current (INaT), which mediates the rising phase of the action potential and is characterized by rapid activation and millisecond-scale inactivation, and the persistent sodium current (INaP), which is notable for not undergoing significant inactivation [10].

## 2. Sodium Channels as a Target for Antiseizure Therapy

Most antiseizure medications (ASMs) have been developed to shift the functional balance of the nervous system toward inhibition over excitation, thereby suppressing or limiting the occurrence of seizures. Although there is currently no curative treatment for epilepsy, the use of ASMs can lead to symptomatic remission or a significant reduction in seizure frequency. This effect arises from diverse mechanisms of action and interactions of these substances with different cellular targets [9]. ASMs exert their therapeutic effects by modulating neuronal excitability through interactions with a broad range of molecular targets, including voltage-gated sodium, calcium, and potassium channels, ligand-gated ion channels such as GABA_A_ and glutamate receptors, synaptic vesicle protein 2A (SV2A), as well as proteins involved in neurotransmitter release and synaptic transmission. Based on their primary molecular targets, ASMs may be broadly classified into sodium channel modulators, calcium and potassium channel modulators, enhancers of GABAergic neurotransmission, inhibitors of glutamatergic neurotransmission, SV2A ligands, and agents acting through multiple complementary mechanisms. This diversity reflects the complex pathophysiology of epilepsy and enables individualized treatment strategies as well as rational combination therapy. Among these targets, VGSCs remain one of the best-characterized and clinically most important molecular targets, making them the focus of several generations of antiseizure medications [11].

Because VGSCs constitute one of the principal molecular targets of ASMs, understanding their physiological function is essential for interpreting the mechanisms of action of sodium channel modulators. In neurons, VGSCs cycle between three main functional states: resting, open, and inactivated. Under resting membrane potential conditions, they remain closed but are ready for rapid activation. Membrane depolarization triggers their rapid opening within milliseconds, allowing an intense influx of sodium ions into the cell and initiating the rising phase of the action potential, which bioelectrically corresponds to the activation of the fast INaT. The channel then transitions into an inactivated state, in which it becomes non-conductive and temporarily unresponsive to further stimuli. Recovery of the channel’s ability to reactivate requires membrane repolarization, which allows it to return to the resting state [12]. In VGSCs, inactivation occurs on two distinct timescales. Fast inactivation develops within milliseconds and is responsible for terminating a single action potential and regulating the refractory period. Slow inactivation occurs much more gradually—over seconds to minutes—and mediates long-term regulation of membrane excitability and the threshold for generating action potentials [13].

Classical ASMs such as phenytoin, carbamazepine, oxcarbazepine (OXC), and lamotrigine preferentially bind to sodium channels in the inactivated state. Structural studies indicate that these drugs bind within the inner pore of VGSCs in a highly conserved region overlapping the local anesthetic (LA) binding site, which is formed mainly by residues located in the S6 helices, particularly the conserved phenylalanine residue in the IVS6 segment. Despite their structural diversity, these ASMs occupy partially overlapping positions within the central cavity and stabilize inactivated channel conformations, thereby producing voltage- and use-dependent inhibition [14]. By stabilizing this state, they prevent the channels from returning to the resting (active) phase, thereby prolonging the neuronal refractory period. These drugs act more strongly at depolarized membrane potentials and during high-frequency firing, allowing selective suppression of pathological seizure activity while minimally affecting normal physiological neuronal function. Their primary target is INaT [9].

Enhancement of slow inactivation of sodium channels by newer ASMs, such as lacosamide (LCM) and eslicarbazepine acetate (ESL), represents an innovative mechanism distinguishing them from classical VGSC blockers [15]. Although both drugs preferentially enhance slow inactivation, their interactions with VGSCs are not identical. Eslicarbazepine, the major active metabolite of eslicarbazepine acetate, is structurally related to carbamazepine and oxcarbazepine and has been reported to interact preferentially with the inactivated state of VGSCs, historically described as an interaction with neurotoxin receptor site 2 [16]. However, currently available evidence suggests that its binding likely involves the same general pore region as classical dibenzazepines, whereas its distinct pharmacological profile results primarily from preferential stabilization of channel conformations associated with slow inactivation rather than from a completely separate binding site [17,18]. These drugs can stabilize sodium channels in a non-conductive state by significantly shifting the voltage dependence of slow inactivation toward hyperpolarized potentials. This effect drastically reduces the pool of channels available for activation at resting potential or during prolonged depolarization, effectively raising the threshold required to generate an action potential. Under pathological conditions, this mechanism selectively suppresses burst firing and synchronous seizure activity while minimally affecting the generation of single, physiological neuronal impulses [13]. Although modulation of VGSC slow inactivation represents the principal mechanism discussed in this section, eslicarbazepine has also been shown to interact with additional molecular targets, including Cav3.2 T-type calcium channels and, more recently, M-type potassium channels, which may further contribute to its antiseizure activity [19,20].

In contrast to eslicarbazepine, the binding mechanism of lacosamide appears to be more complex. Functional studies demonstrated that lacosamide-mediated inhibition requires both an intact pore-associated LA binding region and residue W1538 located in the S2 helix of voltage-sensing domain IV (VSD4), supporting a hybrid VSD4–pore binding mechanism [21,22]. Recent cryo-EM structures of Nav1.7 further identified two lacosamide binding positions: one beneath the intracellular gate (site BIG), which is also occupied by carbamazepine and bupivacaine, and another extending from the central cavity toward the selectivity filter. These findings indicate that lacosamide shares part of the classical pore-binding region while additionally interacting with structurally distinct regions that are not characteristic of older ASMs [23]. Besides its effects on VGSCs, lacosamide has also been proposed to modulate CRMP-2-dependent signaling pathways, although the contribution of this mechanism to its clinical antiseizure efficacy remains under active investigation [24,25].

Additionally, enhancement of slow inactivation is associated with inhibition of the INaP. INaP represents a small, typically 1–2% fraction of the total sodium current, which, unlike the transient component, does not undergo rapid inactivation even during prolonged depolarization. Activated at subthreshold voltages, this current physiologically amplifies synaptic responses and supports rhythmic neuronal firing; however, under pathological conditions, it plays a key epileptogenic role. Excessive INaP leads to pathological network hypersynchrony, generation of burst firing, and maintenance of depolarized plateau potentials [26,27]. This phenomenon is a common feature of many genetic channelopathies caused by mutations (e.g., in SCN1A, SCN2A, SCN3A, and SCN8A) as well as acquired channelopathies, for example following a prolonged seizure state, resulting in a drastically lowered seizure threshold [9]. For this reason, preferential blockade of the INaP by modern drugs, such as cenobamate (CNB), is considered a highly effective therapeutic strategy for controlling seizures in patients with drug-resistant epilepsy [28]. Electrophysiological studies have demonstrated that CNB preferentially inhibits persistent sodium current while exhibiting higher affinity for inactivated VGSCs [10]. Although its precise structural binding site remains less well established than that of lacosamide, recent molecular docking and molecular dynamics studies using an open-state Nav1.5 model suggest that cenobamate binds within a predominantly hydrophobic pocket in the central cavity formed by several S6 segments. Predicted interactions include hydrogen bonding with N932 and hydrophobic contacts with residues such as F1418, F1459, F1463, and I1768, indicating partial spatial overlap with the inner-pore region occupied by classical sodium channel blockers [29]. However, these findings are currently based mainly on computational studies of the cardiac Nav1.5 isoform, and confirmation in epilepsy-relevant neuronal sodium channel isoforms is still required. In addition to preferential inhibition of persistent sodium current, cenobamate also enhances GABAergic neurotransmission through positive allosteric modulation of GABA_A_ receptors via a binding site distinct from the benzodiazepine site, providing an additional mechanism that may contribute to its remarkable clinical efficacy [10,30].

**Figure 1:**
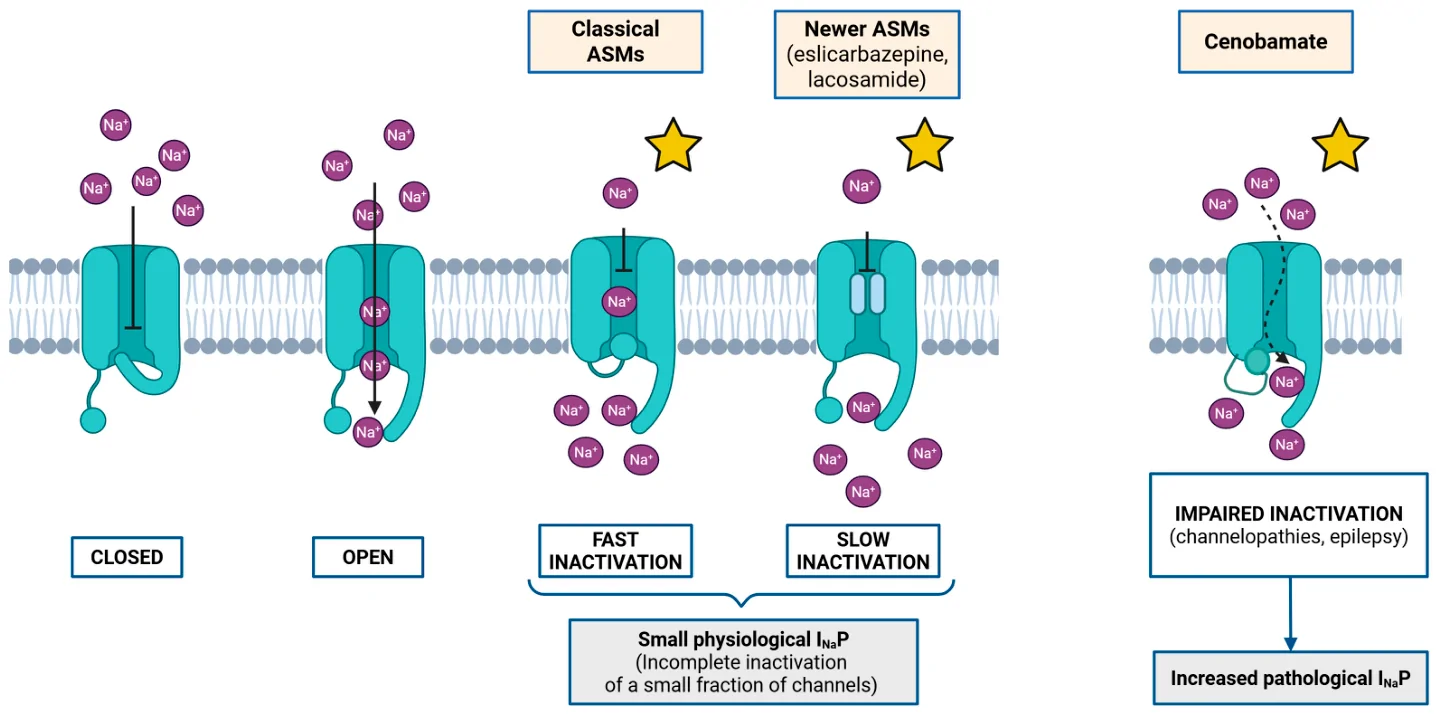
Voltage-gated sodium channel states and antiseizure medication (ASMs) targets. Voltage-gated sodium channels (VGSCs) transition between closed, open, fast-inactivated, and slow-inactivated states. Fast and slow inactivation are grouped to highlight that incomplete inactivation of a small fraction of channels generates a physiological persistent sodium current (I_Na_P). Impaired inactivation, observed in channelopathies and in certain forms of epilepsy, leads to an increased pathological I_Na_P, contributing to neuronal hyperexcitability. Classical ASMs primarily act by stabilizing the fast-inactivated state, whereas newer agents such as eslicarbazepine and lacosamide preferentially stabilize the slow-inactivated state. Cenobamate is associated with modulation of impaired inactivation and reduction of I_Na_P. 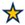
—indicates drug binding sites (targets) of ASMs. Created in BioRender (BioRender Inc., Toronto, ON, Canada). Budzyńska, B. (2026) https://BioRender.com/bnjdk50 (accessed on 23 July 2026) [31].

Taken together, current evidence indicates that newer sodium channel-targeting ASMs do not necessarily occupy entirely distinct receptor regions compared with classical sodium channel blockers. Instead, their binding sites appear to partially overlap within the conserved inner pore, while differences in binding orientation, additional interactions with voltage-sensing or selectivity-filter regions, and preferential stabilization of specific channel conformational states underlie their distinct pharmacological profiles [14,22,23,29]. The mechanisms of action of antiseizure medications targeting voltage-gated sodium channels are summarized in Figure 1.

## 3. Novel Sodium Channel-Modulating Antiseizure Medications

### 3.1. Eslicarbazepine

**Figure 2:**
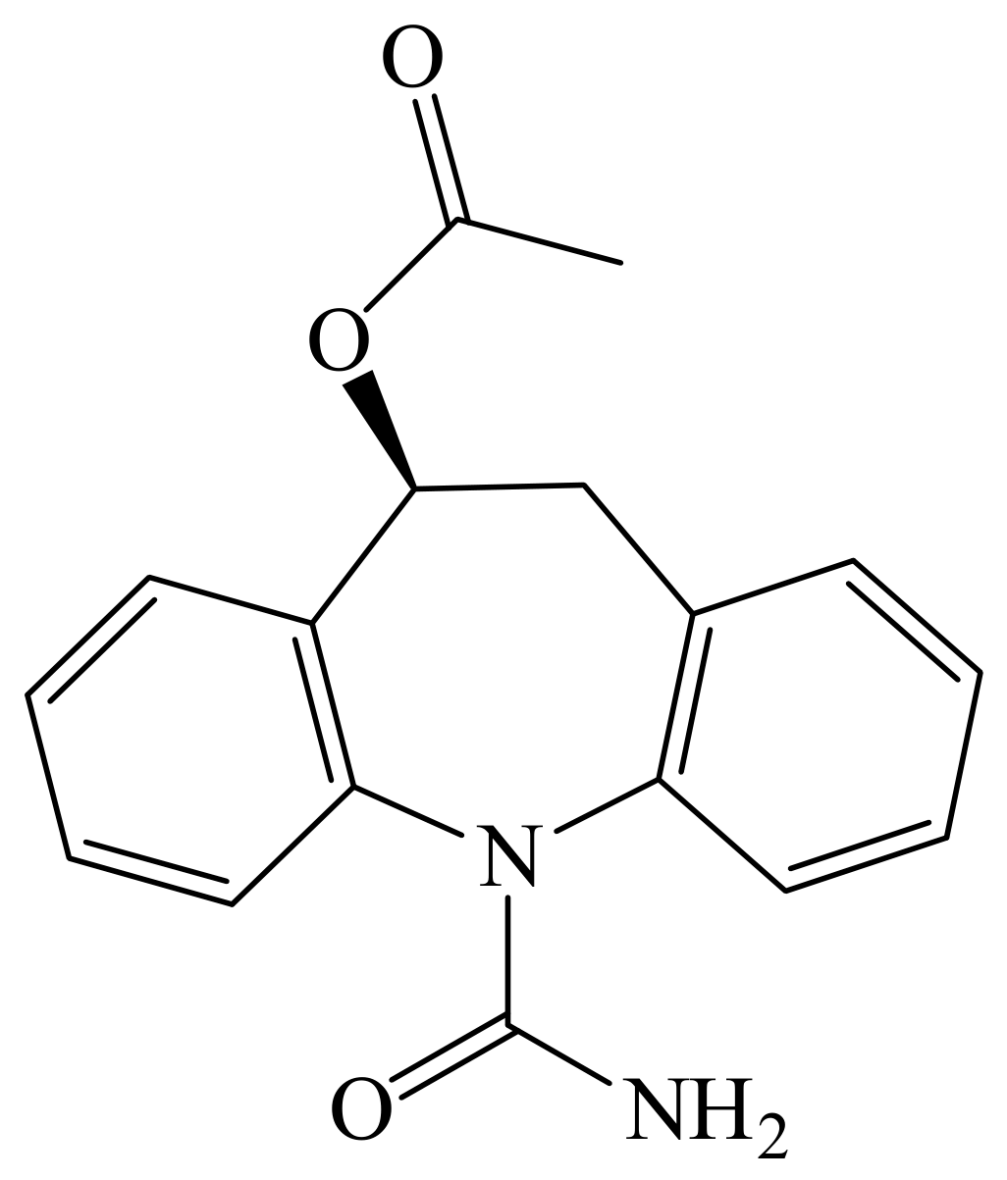
Structure of eslicarbazepine (ESL).

Eslicarbazepine acetate (ESL), a third-generation antiseizure drug from the dibenzazepine family (Figure 2), is a prodrug that undergoes rapid and almost complete conversion in the body to its active metabolite: (S)-licarbazepine (eslicarbazepine) [32]. This substance has been approved for use in both monotherapy and adjunctive treatment of focal seizures and was introduced into clinical practice under the trade names ZEBINIX^®^ in Europe and APTIOM^®^ in the United States [33,34]. Although the mechanism of action of ESL, like that of older-generation drugs, involves an effect on sodium channels, it exhibits significant differences. This drug stabilizes the slow inactivation state of VGSCs, which allows for the inhibition of the activity of neurons generating epileptic discharges while sparing the normal function of neurons [17,18,20,35].

Although enhancement of VGSC slow inactivation represents the principal antiseizure mechanism of eslicarbazepine, accumulating evidence indicates that its pharmacological profile extends beyond sodium channel modulation. Eslicarbazepine has been shown to inhibit Cav3.2 T-type calcium channels with considerably greater potency than carbamazepine while exerting little or no effect on P/Q-type calcium channels, a property that may further reduce neuronal burst firing and network synchronization [18,19]. More recently, experimental studies have demonstrated that eslicarbazepine also enhances M-type potassium currents (I_K(M)_) in hippocampal neurons, thereby stabilizing membrane excitability and limiting repetitive neuronal firing [20]. These complementary mechanisms may contribute to the overall antiseizure efficacy of ESL, particularly under conditions of neuronal hyperexcitability [19,20].

A significant advantage of such a profile is a lower risk of interactions with other drugs and a weaker effect on hepatic enzymes compared to first-generation dibenzazepines [35,36]. The pharmacokinetic properties of the drug, including a long half-life, enable convenient once-daily administration. ESL is characterized by high bioavailability and is metabolized in a way that avoids the formation of certain toxic metabolites typical of older drugs in this group [32,37]. Available data indicate that the drug is generally well tolerated across age groups, including the elderly and children [34,38]. Based on information from clinical trials, eslicarbazepine acetate is indicated for the treatment of focal seizures, which may or may not secondarily generalize [37]. The drug can be used as adjunctive therapy in a wide spectrum of patients, including adults and the pediatric population [34,36]. It is also approved for use as a monotherapy in adults [32,33]. Studies confirm that regular intake of the drug at therapeutic doses leads to a significant reduction in seizures and improvement in treatment response rates compared to placebo [34,37]. Similarly to other pharmaceuticals, the use of ESL may be associated with the occurrence of adverse events. The most frequently reported complaints include nervous system symptoms such as dizziness, somnolence, or visual disturbances, as well as gastrointestinal complaints [37,38]. It is worth noting the possibility of lowered sodium levels in the body (hyponatremia), which is a known phenomenon in this group of drugs and may occur more frequently in elderly patients [38,39]. Nevertheless, the overall safety profile of the drug is assessed as favorable, and serious adverse events occur relatively rarely [35,36].

#### 3.1.1. Eslicarbazepine Acetate as Monotherapy

Eslicarbazepine acetate, a third-generation dibenzazepine approved for once-daily administration, represents a valuable option for monotherapy due to its linear pharmacokinetics and low potential for interaction [32]. The use of monotherapy is especially critical in elderly patients, who are more vulnerable to adverse events and polypharmacy. In this population, ESL has demonstrated a consistent safety profile. However, specific attention to hyponatremia is required, as its incidence is higher in the elderly (6.7%) compared to the non-elderly population (1.5%) [38]. While most new antiseizure drugs are initially approved for adjunctive therapy, the regulatory pathway for monotherapy approval involves rigorous testing. In the United States, the FDA allows for “conversion to monotherapy” trial designs utilizing historical controls, whereas other regions often require active-control comparisons.

A pivotal study supporting the efficacy of ESL as monotherapy was conducted by Jacobson et al. 2015 [32]. This Phase III, randomized, double-blind, multicenter, historical-control study evaluated the safety and efficacy of ESL monotherapy in adults with partial-onset seizures not well controlled by 1 or 2 current ASMs. The study involved a conversion to monotherapy design. Eligible patients were randomized in a 1:1 ratio to receive ESL 1600 mg/day (*n* = 196) or ESL 1200 mg/day (*n* = 185) once daily. After a titration period, background ASMs were withdrawn over 6 weeks, followed by a 10-week monotherapy maintenance period. The primary endpoint was the exit rate (proportion of patients meeting specific exit criteria signifying worsening of seizure control) by day 112 compared to a historical control threshold of 65.3%. The study demonstrated that exit rates for both ESL doses were significantly lower than the historical control threshold. The Kaplan–Meier estimated exit rates were 12.8% for the 1600 mg/day group and 15.6% for the 1200 mg/day group (upper 95% confidence limits were well below the 65.3% benchmark, *p* < 0.0001). These results confirmed that ESL monotherapy is effective in maintaining seizure control following the withdrawal of concomitant ASMs. Furthermore, the median reduction in standardized seizure frequency between baseline and the 16-week double-blind period was 41.3% for the 1600 mg group and 38.3% for the 1200 mg group [32].

Complementing the clinical trial data, real-world evidence regarding ESL monotherapy was analyzed by Fernández-Anaya et al. 2023 [33] in a systematic review and meta-analysis of observational studies. This analysis included 17 studies comprising 2122 patients treated with ESL monotherapy (either as initial monotherapy or after switching from other drugs). The results provided robust data on effectiveness and retention in clinical practice. The pooled retention rates for ESL monotherapy were high: 78.6% (95% CI: 72.3–84.3%) at 6 months and 65.5% (95% CI: 54.4–75.8%) at 12 months. Regarding efficacy, the pooled seizure freedom rates were 56.6% (95% CI: 45.4–67.4%) at 6 months and 45.4% (95% CI: 33.3–57.8%) at 12 months. The study also highlighted that the retention rate was slightly higher in patients receiving ESL as initial monotherapy compared to those switched to ESL due to lack of efficacy with previous treatments. The safety profile in this real-world setting was consistent with clinical trials: a pooled proportion of patients reporting at least one adverse event was 27.2%, and the rate of discontinuation due to adverse events was 8.9%. These findings support the utility of ESL as a viable monotherapy option in routine clinical practice for adult patients with focal epilepsy [33].

**Tolerability and side effects of ESL monotherapy**

Data analysis reveals a favorable tolerability profile for ESL as a monotherapy drug. Adverse events were generally reported as mild or moderate in intensity [32]. In a comprehensive meta-analysis of real-world clinical practice studies, adverse events were recorded in 27.2% of patients treated with ESL monotherapy, among which 8.9% decided to discontinue therapy due to side effects [33]. The most frequently reported adverse events were dizziness, somnolence, nausea, headache, and diplopia. Additionally, specific attention is drawn to asymptomatic hyponatremia, particularly in elderly patients, although severe cases remain rare [37,38].

#### 3.1.2. Eslicarbazepine Acetate as Add-On Therapy

Significant evidence for the efficacy of ESL as add-on therapy in adults comes from large Phase III clinical trials. Sperling et al. 2015 [37] presented results from a randomized, double-blind, placebo-controlled study involving patients aged 16 and older who had uncontrolled partial-onset seizures despite treatment with 1–2 antiseizure drugs. Patients were randomized to receive placebo, ESL 800 mg, or ESL 1200 mg once daily. The study demonstrated that ESL significantly reduced standardized seizure frequency (SSF). The median reduction in SSF over the 12-week maintenance period was 33.8% for the 800 mg group and 37.3% for the 1200 mg group, compared to established baselines. Furthermore, the responder rate (defined as ≥50% reduction in seizure frequency) was 33.6% for ESL 800 mg and 43.1% for ESL 1200 mg. Discontinuation rates due to adverse events increased with the dose (13.4% for 800 mg and 27% for 1200 mg), with dizziness and diplopia being the most common reasons [37].

In the pediatric population, the utility of ESL as adjunctive therapy has also been confirmed. Kirkham et al. 2020 [34] conducted a Phase III study in children with refractory focal-onset seizures. The study showed that ESL (target dose of 30 mg/kg/day) resulted in a statistically significant reduction in standardized seizure frequency compared to placebo (*p* < 0.001) in patients aged 2 to 18 years. This confirms that ESL is a viable add-on option for the pediatric population, providing a significant therapeutic benefit in reducing seizure burden [34].

Real-world data further support the use of ESL in diverse clinical scenarios, including drug-resistant epilepsy in children. Nyakeri et al. 2025 [36] analyzed the use of ESL in 50 children with drug-resistant epilepsy, most of whom were on multiple medications (mean of 2.2 concurrent ASMs). The study reported retention rates of 64% at 1 year and 52% at 2 years. Efficacy analysis showed that 41.2% of patients were responders (≥50% seizure reduction) at 3 months, and 11.8% achieved seizure freedom. The study highlighted that ESL is generally well-tolerated as an add-on, with fatigue and dizziness being the most reported adverse effects [36].

Interesting results regarding the optimization of polytherapy were presented by Shim et al. 2025 [40]. This study focused on patients who were switched from OXC to ESL, often to reduce pill burden or manage side effects while maintaining add-on efficacy. The switch was conducted overnight (1:1 dose ratio). The study found that switching was safe, with no serious adverse events reported. Tolerability was high, and serum sodium levels remained stable or improved in some cases, suggesting that ESL can be successfully integrated into complex therapeutic regimens or used to simplify treatment in patients previously treated with related dibenzazepines [40].

Safety in adjunctive therapy remains a critical consideration, particularly for vulnerable groups. Magalhães et al. 2021 [38] analyzed pooled data from clinical studies and emphasized that while the overall safety profile is favorable, the incidence of hyponatremia is age-dependent. In elderly patients (>65 years) treated with ESL (mostly as add-on), hyponatremia was reported in 6.7% of patients compared to 1.5% in the non-elderly. This indicates the need for careful monitoring of sodium levels when ESL is added to the therapeutic regimen of older patients [38].

**Tolerability and side effects of ESL add-on therapy**

ESL add-on therapy is generally well tolerated, though the incidence of adverse events (AEs) often follows a dose-dependent pattern. In a pivotal Phase III study involving adults, discontinuation rates due to AEs were 13.4% for the 800 mg dose and rose to 27.0% for the 1200 mg dose. The most frequently reported adverse events included dizziness, somnolence, nausea, headache, and diplopia [37]. In the pediatric population, the safety profile remains consistent with adult data. Kirkham et al. 2020 [34] reported that treatment-emergent AEs occurred in 70.3% of ESL-treated children compared to 52.8% in the placebo group, with headache, somnolence, and nasopharyngitis being predominant [34]. Real-world data from tertiary care centers indicate that while fatigue and behavioral changes may occur, the discontinuation rate due to side effects in children is approximately 12% [36].

Specific consideration is needed for elderly patients. A pooled analysis revealed that while general neurological AEs like dizziness are common across all ages, the incidence of hyponatremia is significantly higher in the elderly (6.7%) compared to non-elderly patients (1.5%) [38]. Although generally manageable, rare cases of severe hyponatremia leading to seizure worsening have been documented, highlighting the need for monitoring [39]. Conversely, transitioning from twice-daily OXC to once-daily ESL in pediatric patients has been shown to be safe and well-tolerated, with no serious adverse events and maintenance of stable serum sodium levels [40].

### 3.2. Lacosamide

**Figure 3:**
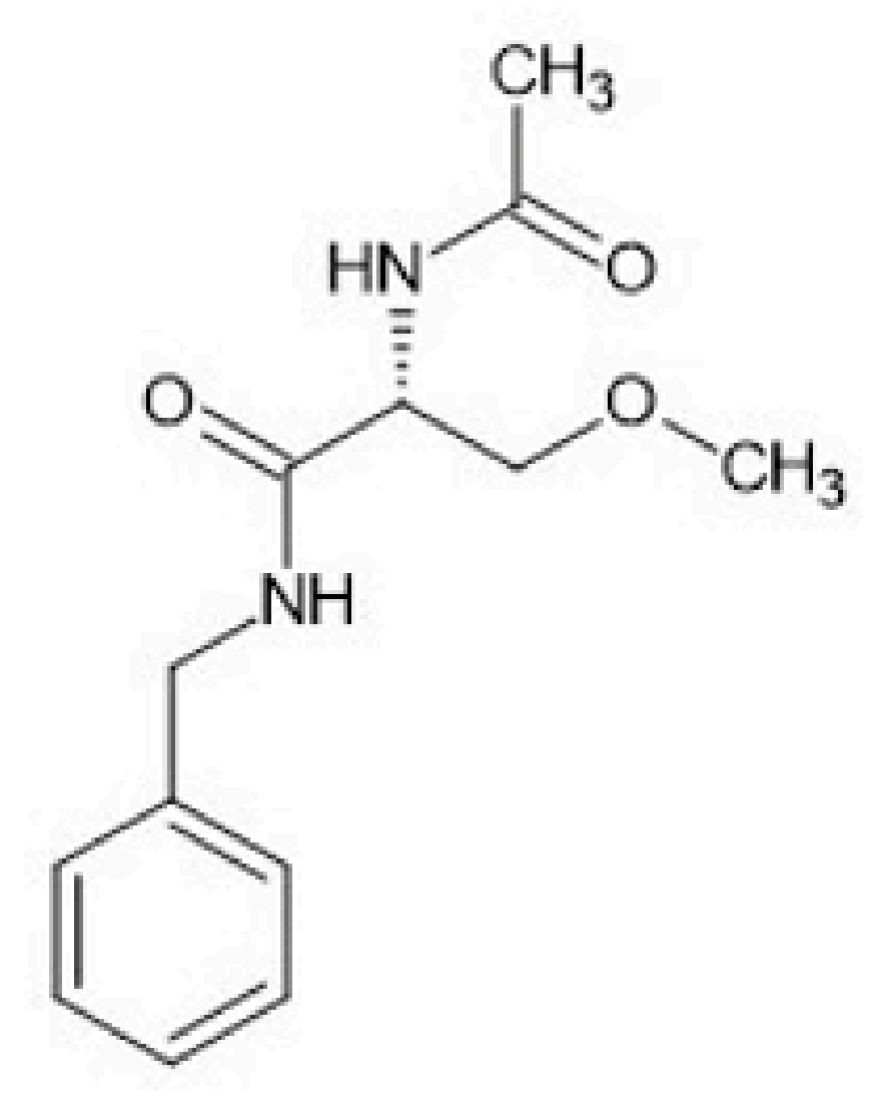
Structure of lacosamide (LCM).

Lacosamide (LCM), the R-enantiomer of 2-acetamido-N-benzyl-3-methoxypropionamide, is a novel antiseizure medication (Figure 3) that has been studied as both mono- and adjunctive therapy with good clinical response [41]. The new third-generation drug, introduced into clinical practice in 2008, exhibits a different mechanism of action from conventional sodium channel blockers. Whereas classical ASMs primarily enhance the fast inactivation of voltage-gated sodium channels, lacosamide selectively enhances their slow inactivation [13,42,43].

Lacosamide specifically targets abnormal currents associated with slow sodium channel inactivation, rather than affecting the fast channel inactivation that occurs in healthy neurons [44]. Carbamazepine, a known ASM, does not affect slow sodium channel inactivation, whereas ASMs exert only minimal effects [45]. Lacosamide differs from classical sodium channel blockers not only in its selective enhancement of slow VGSC inactivation but also in its proposed interaction with collapsin response mediator protein-2 (CRMP-2), a phosphoprotein involved in axonal growth, neuronal differentiation, and synaptic plasticity. Experimental studies suggest that lacosamide may modulate CRMP-2 function, thereby limiting pathological axonal sprouting and the formation of aberrant neuronal connections that are thought to contribute to epileptogenesis. Although CRMP-2 has been proposed as an additional molecular target of lacosamide, the contribution of this interaction to its clinical antiseizure efficacy remains controversial and requires further investigation [22,24,25].

An important advantage of LCM is its low potential for drug–drug interactions, as it is eliminated primarily by renal excretion and does not undergo significant metabolism by the cytochrome P450 enzyme system [46]. It is known that the drug is characterized by linear kinetics and a half-life of 13 h, which allows for twice-daily administration [47]. The pharmacokinetics of LCM are not significantly influenced by age (>65 years), sex, race, or CYP2C19 polymorphism [48]. In accordance with information presented by Thomas et al. 2007 [49], LCM is completely absorbed from the gastrointestinal tract after oral administration, and food does not influence the process [50]. It is also significant that LCM does not interact with other important drugs such as carbamazepine CBZ, valproic acid (VPA), metformin, digoxin, or oral contraceptives [51,52,53].

Based on the information presented by the European Medicines Agency, lacosamide, marketed under the trade name VIMPAT^®^, is approved for use in both monotherapy and adjunctive therapy for the treatment of partial-onset seizures, with or without secondary generalization, in patients with epilepsy aged 2 years or older. VIMPAT^®^ may also be prescribed alongside other ASMs to treat primary generalized tonic-clonic seizures (commonly involving loss of consciousness) in patients aged 4 years and older who have idiopathic generalized epilepsy, a form of epilepsy believed to have a genetic origin [54]. Similar indications have been approved by the U.S. Food and Drug Administration (FDA) when it approved the drug for use in the United States of America. Since its approval in 2008, lacosamide has been indicated as adjunctive therapy for the treatment of partial-onset seizures in adults. Subsequent label expansions included approval for monotherapy in patients aged 4 years and older, extension of its use to the pediatric (≥4 years) population, and adjunctive treatment of primary generalized tonic-clonic seizures [55].

Similarly to other drugs, LCM is associated with adverse effects. Based on a systematic review and meta-analysis presented by Yang et al. 2021 [56], which included 83 studies involving more than 12,000 patients, the following incidence rates of adverse events (AEs) were reported: overall AEs, 38.7%; serious AEs, 6.5%; treatment discontinuation due to AEs, 10.8% and the most common AEs were as follows: dizziness (15.7%), sedation (15.8%), fatigue (9.4%), and nausea/vomiting (9.3%) [56]. According to the 2024/2025 update of the FDA prescribing information, LCM is associated with common adverse reactions, including diplopia, headache, dizziness, nausea, and somnolence. The updated safety information also highlights the risk of suicidal ideation and behavior; cardiac conduction abnormalities; syncope; and DRESS (Drug Reaction with Eosinophilia and Systemic Symptoms) hypersensitivity reactions [57].

#### 3.2.1. Lacosamide as Monotherapy

There is a limited number of studies based on LCM as monotherapy. Long-term studies have led to the approval of LCM for monotherapy in 2014. The U.S. FDA approved this indication primarily based on the historical-controlled trial conducted by Wechsler et al. 2014 [58], supported by evidence from prospective and retrospective studies, as well as data presented in conference abstracts [48,59,60,61].

Wechsler et al. 2014 [58] conducted the ALEX-MT trial, a multicenter, double-blind, historical-controlled study evaluating conversion from one or two stable ASMs to oral lacosamide monotherapy in patients aged 16–70. Eligible participants (experiencing 2–40 focal seizures per 28 days) were randomized in a 3:1 ratio to receive either 400 mg/day (*n* = 319) or 300 mg/day (*n* = 106) LCM. The study consisted of a 3-week titration phase followed by a 16-week maintenance phase (with ASM withdrawal and monotherapy periods). In the full analysis set (*n* = 284), 30.0% of patients receiving 400 mg/day met the predefined exit criteria by day 112, a value significantly below the historical control benchmark of 65.3%, thereby demonstrating the efficacy of lacosamide monotherapy. The mean time to meeting the exit criteria was 45 days, whereas the median duration of monotherapy was 71 days. Clinical improvement was reported by more than 74% of patients on PGIC and CGIC scales. Furthermore, a post hoc analysis showed seizure reductions of ≥50%, ≥75%, and complete seizure freedom (100%) in 60.7%, 34.3%, and 14.9% of patients, respectively [58].

In 2017, Baulac et al. 2017 [62] provided important findings from a study that assessed the efficacy, safety, and tolerability of LCM as a first-line monotherapy in patients with newly diagnosed epilepsy, following the guidelines set by the EMA and the International League Against Epilepsy (ILAE). This randomized, double-blind, non-inferiority trial included patients aged 16 and older with newly diagnosed epilepsy from 185 centers across Europe, North America, and the Asia-Pacific region. Participants were randomly assigned to receive either LCM or controlled-release CBZ (carbamazepine-CR) as monotherapy. Treatment was initiated at 100 mg/day for LCM and 200 mg/day for carbamazepine-CR as monotherapy. After a one-week stabilization period, patients entered a 6-month assessment phase. If seizures occurred, doses were increased to higher target levels, and the process was repeated. Those who remained seizure-free for six months continued into a maintenance phase. The primary endpoint was the proportion of patients achieving 6-month seizure freedom. Non-inferiority was predefined as an absolute treatment difference of −12% or a relative difference of −20% between the treatment groups. The trial was registered at ClinicalTrials.gov (NCT01243177). The trial was conducted between April 2011 and August 2015, enrolling 888 patients who were randomly assigned to receive either LCM or carbamazepine-CR. In the full analysis set, 444 patients received lacosamide and 442 received carbamazepine-CR, whereas 408 and 397 patients, respectively, were included in the per-protocol analysis. At six months of treatment, 74% of patients receiving lacosamide and 70% of the carbamazepine-CR group were seizure-free. Kaplan–Meier analysis estimated seizure freedom in 90% of lacosamide-treated and 91% of carbamazepine-CR-treated patients, with similar results in the per-protocol group. Adverse events occurred in 74% of LCM patients and 75% of those treated with carbamazepine-CR. Serious adverse events, as well as treatment discontinuations, occurred slightly more frequently in the carbamazepine-CR group. Overall, lacosamide met the predefined criteria for non-inferiority, suggesting that it represents an effective and well-tolerated first-line monotherapy option for newly diagnosed adult epilepsy patients [62].

Equally important findings were presented by Lattanzi et al. 2015 [59], who evaluated the long-term efficacy of LCM monotherapy in patients with focal seizures over a 1-year follow-up period. The prospective study was a conversion add-on therapy from LCM to LCM monotherapy (350–400 mg/day, 1 year, *n* = 58). The main outcomes assessed were one-year seizure freedom following withdrawal of previous ASMs and the retention rate of LCM as monotherapy. Seizure freedom was achieved in 55.2% of patients, whereas 8.6% experienced at least a 75% reduction in seizure frequency. Among patients who were previously treated with one or two ASMs, 69.2% maintained seizure freedom, compared with 26.3% of those who had received three or more prior ASMs. The overall 1-year retention rate of LCM monotherapy was 63.8% [59].

**Tolerability and side effects of LCM monotherapy**

Available clinical evidence indicates that LCM is generally well tolerated when used as monotherapy. In the pivotal historical-controlled trial, most adverse events were mild to moderate in severity [58]. Treatment-emergent adverse events (TEAEs) were reported in 84.5% of patients (in the historical-controlled study), although only 16.9% discontinued treatment because of adverse events. The most common adverse events were dizziness, headache, nausea, convulsions, somnolence, and fatigue [48,58].

#### 3.2.2. Lacosamide as Add-On Therapy

Lacosamide is widely used as add-on therapy for patients with focal-onset seizures, with or without secondary generalization [63,64]. It has been approved for this indication in both the U.S. and European Union for adults and children aged 4 years and older. In individuals aged 16 years and older, adding oral LCM to existing ASMs has been shown to effectively reduce seizure frequency during short-term treatment (up to 18 weeks), with this benefit maintained over extended treatment (up to 8 years) [65].

The beneficial effects of adjunctive LCM therapy have been demonstrated in various patient populations. The most commonly studied combinations of LCM with other ASM drugs are LCM and VPA, levetiracetam (LEV), and CBZ, as well as studies in brain tumor-related epilepsy (BTRE) and Lennox-Gastaut syndrome. An interesting observation was reported by Ruffolo et al. 2018 [66], who indicated that LCM alone had no significant effect on GABAergic neurotransmission. In contrast, its combination with levetiracetam significantly reduced inward GABA currents, suggesting a pharmacodynamic interaction between the two drugs.

Interesting results were presented by Moosavian and Moosavian (2024) [67], who focused on LCM as an adjunctive therapy for drug-resistant absence epilepsy. Their case series included four children (three boys and one girl), aged 4–10 years at treatment initiation. All patients had normal neurodevelopment and no prior hospitalizations before starting antiseizure medication. One child, who had experienced staring spells accompanied by blinking since the age of two, was suspected to have Jeavons syndrome, whereas the remaining patients presented with frequent absence seizures (over 20 daily) along with 1–2 generalized seizures. All patients were receiving multiple medications, and their EEGs revealed generalized 3 Hz spike-wave patterns. Following introduction of LCM, all patients became seizure-free within one week at the target dose. Subsequently, LEV and ethosuximide were gradually withdrawn, while VPA and LCM were maintained. Lacosamide has been shown to be effective primarily in the treatment of focal-onset seizures, which are the most prevalent seizure type in epilepsy. Research from clinical trials indicates that when added to existing treatment regimens, LCM can substantially decrease seizure frequency and enhance seizure management in patients with drug-resistant focal seizures. A comprehensive Cochrane systematic review by Babar et al. 2021 [68] evaluated the efficacy and tolerability of LCM as adjunctive therapy for drug-resistant focal epilepsy. The evaluation was based on placebo-controlled studies and doses of 200–600 mg per day. Among the participants, one study was aimed at children. The length of the trials varied between 24 and 26 weeks. Each study employed proper randomization techniques and maintained a double-blind design. Pooled analysis demonstrated that patients receiving LCM were 1.79 times more likely to achieve a ≥50% reduction in seizure frequency than those receiving placebo. Furthermore, the likelihood of achieving seizure freedom was significantly higher with LCM (risk ratio, 2.27). However, treatment discontinuation due to adverse events occurred more frequently among patients receiving LCM (risk ratio of 1.57) [68].

Similarly important study results were presented by Jin et al. (2023) [69], who evaluated the efficacy and tolerability of LCM as an add-on therapy in patients with focal-onset seizures. In this single-center prospective observational study, 106 patients aged 16 years and older were consecutively enrolled. Based on clinical judgment, all participants received LCM as an adjunctive therapy. Data on seizure frequency, adverse events, and retention rates were collected at 3 and 6 months following the initiation of LCM. Scientists noticed that response rates after 3 and 6 months were 53.3% and 70.4%, respectively, whereas freedom from seizures was reached at 19% and 26.5%, respectively. After 3 months of LCM introduction, retention rates were 99.1%, whereas 93.3% was reached after 6 months. It is also important that only 16.98% of dizziness and 6.6% of sedation were observed as adverse effects [69].

A significant part of studies based on LCM includes its activity against BTRE. Since the drug is often used in add-on therapy, its use in this case is justified. Interesting study results were presented by Maschio et al. 2017 [64] who decided to compare LCM and LEV. The studies were performed on 25 patients treated with LCM with a historical control group (*n* = 19) treated with LEV as an add-on. Out of 25 patients, 12 had high-grade and 13 low-grade gliomas. During follow-up, 13 received chemotherapy, 3 radiotherapy, and 5 showed disease progression. Seizure types included simple partial (9), complex partial (8), and secondary generalized (8). Fifteen were on monotherapy and 10 on polytherapy. Lacosamide was titrated up to 400 mg/day (mean 300 mg/day). Five patients discontinued (4 poor adherence, 1 inefficacy). Among the 22 evaluable patients, 7 became seizure-free, 12 achieved ≥50% reduction, 2 remained stable, and 1 worsened. Comparison of responder rates between LCM (22 patients) and LEV (19 patients) showed no significant difference (*p* = 0.31). An analysis of the results indicated that LCM was a better add-on ASM than LEV [64].

Slightly different results were obtained by Acar and Aras in 2018 [70], who compared LCM and LEV in patients with partial-onset seizures. The study included two groups: 30 patients treated with LEV and 28 patients treated with LCM. Observation and measurement were conducted 6 months before and 3 and 6 months after add-on therapy. The obtained results did not reveal significant differences between the two analyzed groups. This fact indicates similar effectiveness of both drugs. High activity of LCM was similar to well-established LEV, which highlights the significance of LCM [55]. In 2025, Bhaumik et al. 2025 [71] conducted a comparative analysis of LCM efficacy and treatment characteristics in patients with epilepsy. The analysis included 685 epilepsy patients (43.94% with generalized tonic-clonic seizures (GTCS) and 56.06% with focal-onset seizures (FoS) who were treated with LCM for at least 12 weeks. The following doses were used: 50 mg, 100 mg, 150 mg, or 200 mg. In 56.50% of patients, LEV was used as co-prescribed anti-seizure medications. After 12 weeks of treatment, the mean frequency of GTCS decreased from 3 to 1 (*p* < 0.0001), with 52.2% of patients seizure-free and a responder rate of 66.8%. The combination of LCM with LEV yielded roughly double the responder rate compared to LCM with other ASMs. In FoS, seizure freedom was reached in 47.7% of patients, with a 58.9% responder rate; as monotherapy, LCM achieved a 63% responder rate. Overall, LCM was well tolerated in both groups, and no new safety issues emerged during the study [71].

**Tolerability and side effects of LCM add-on therapy**

LCM add-on therapy is generally well tolerated across different patient populations. In patients with brain tumor–related epilepsy, only one out of 14 patients discontinued treatment due to adverse effects, and no additional safety concerns were reported [72]. In a Middle Eastern study of 104 patients, the retention rate was 88%, with dizziness, somnolence, diplopia, and gastrointestinal symptoms being the most frequent adverse events [73]. An Australian multicenter analysis involving 310 patients identified factors linked to better tolerability, including a history of focal-to-bilateral tonic-clonic seizures, lower seizure frequency at baseline, and reduction of concomitant ASMs after starting LCM [74]. In pediatric patients, the most frequently reported adverse events included dizziness, ataxia, nausea, and vomiting; however, two cases of status epilepticus were noted shortly after treatment initiation, marking the first such reports in pediatric populations [75].

### 3.3. Cenobamate

**Figure 4:**
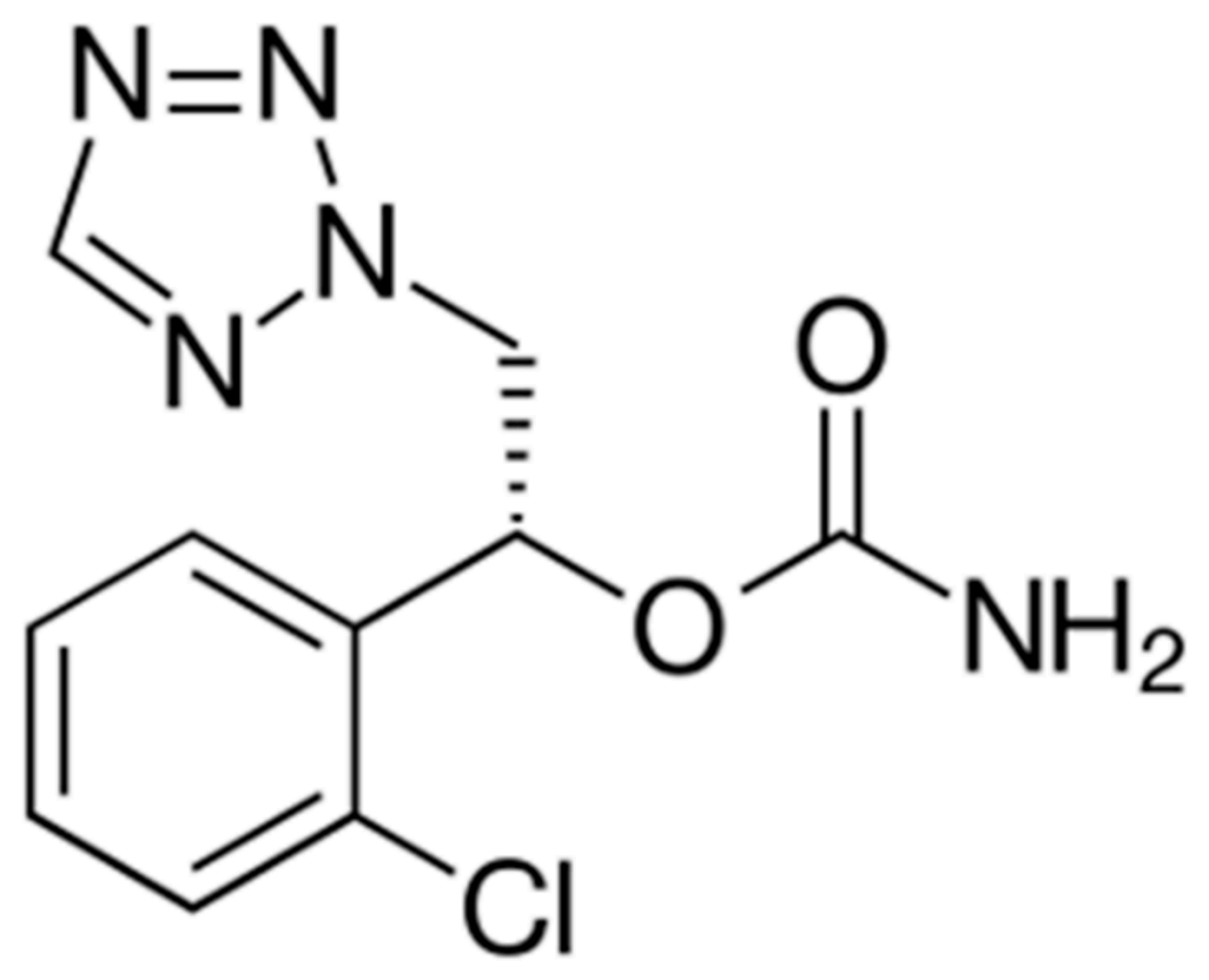
Structure of cenobamate (CNB).

Cenobamate (CNB), chemically known as [(1R)-1-(2-chlorophenyl)-2-(imidazol-1-yl)ethyl] carbamate (Figure 4), is a novel ASM that has shown significant efficacy in both adjunctive and monotherapy treatment for adults with uncontrolled focal seizures [76,77,78]. CNB was approved for clinical use in 2019, representing a third-generation antiseizure drug that offers new options for patients with refractory epilepsy [28,79].

CNB demonstrates a unique dual mechanism that involves selective inhibition of INaP and positive allosteric modulation of GABA_A_ receptors, which together contribute to its anticonvulsant efficacy [28,80], as described in detail below.

Pharmacokinetically, CNB exhibits favorable properties, including high oral bioavailability and a long half-life of approximately 50 to 60 h, enabling convenient once- or twice-daily dosing suitable for long-term treatment [28,78]. CNB is metabolized mainly through glucuronidation and oxidation by cytochrome P450 enzymes (CYP2C19 and CYP3A4), which may contribute to potential drug–drug interactions and, therefore, require close monitoring [77,81]. Notably, a well-adjusted dosing schedule is crucial to minimize risks of serious adverse events, including DRESS [76,78].

CNB is marketed under the trade name XCOPRI^®^ in the United States (SK Life Science, Inc.; Paramus, NJ, USA) and as ONTOZRY^®^ in the European Union (Angelini Pharma S.p.A; Rome, Italy) and is approved as adjunctive therapy for focal-onset seizures in adults aged 18 years and older [76,82,83]. Its regulatory approval was supported by robust randomized controlled trials that demonstrated superior efficacy and acceptable safety profiles compared to placebo [28,76]. Real-world evidence further substantiates its clinical benefit and manageable tolerability profile. Ongoing research continues to explore its optimal clinical use across diverse epilepsy populations [77,78,84].

**Dual mechanism of action of CNB**

CNB exhibits a unique dual mechanism of action that distinguishes it from other ASMs and may explain its high efficacy observed in clinical trials [28].

CNB acts on VGSCs with a pronounced inhibitory effect on the persistent sodium current (INaP), rather than the transient sodium current (INaT) [10]. This property enhances inactivation kinetics and stabilizes hyperexcitable neuronal membranes, reducing repetitive neuronal firing. Unlike classical sodium channel blockers (such as carbamazepine, lamotrigine, or phenytoin), CNB preferentially blocks INaP while allowing transient currents—essential for normal neuronal conduction—to remain intact [11,80]. This selectivity may preserve the physiological inhibitory function of fast-spiking GABA-ergic interneurons, minimizing cognitive and sedative side effects [85,86]. The importance of INaP in regulating neuronal excitability has been demonstrated, along with evidence indicating that inhibition of this current can significantly reduce seizure propagation [86,87].

In addition to sodium channel blockage, CNB serves as a positive allosteric modulator of both synaptic and extrasynaptic GABA_A_ receptors [30,88]. It has been shown to enhance inhibitory transmission by simultaneously increasing phasic and tonic GABA-ergic currents [30]. Importantly, this modulation occurs independently of the benzodiazepine (BDZ) binding site and cannot be antagonized by flumazenil, suggesting that CNB acts through distinct receptor subunits [30,88,89]. The presented activity leads to the potentiation of inhibitory signaling without directly engaging traditional BDZ-sensitive sites, reducing the risk of tolerance and dependence associated with long-term BDZ use. By enhancing tonic inhibition in hippocampal and cortical neurons, CNB contributes to the restoration of excitatory/inhibitory balance disrupted in the epileptic focus [30,80,88].

The combination of selective blockade of the INaP current and enhancement of both phasic and tonic GABA-ergic inhibition creates a synergistic effect, limiting neuronal hyperexcitability while amplifying inhibitory tone [10,30,80]. The concentration-dependent inhibitory potency for INaP (IC_50_ ≈ 53 µM) and facilitation of GABA_A_ receptor activation (EC_50_ ≈ 42–194 µM) further support the efficacy of CNB’s dual mechanism at therapeutic plasma concentrations [30]. This combined mode of action—distinct from previously known antiseizure drugs—likely underlies CNB’s superior clinical efficacy in reducing seizure rates (approaching 20% in patients with uncontrolled focal epilepsy) [10,30,76,90].

#### 3.3.1. Cenobamate as Monotherapy

CNB is currently approved and widely studied as adjunctive therapy; however, emerging evidence explores its use as monotherapy. Early-phase pharmacokinetic and pharmacodynamic modeling, along with retrospective claims data, indicate that CNB monotherapy at doses of 100 mg to 200 mg daily can lead to CNB plasma levels comparable to those seen in effective adjunctive treatments [91]. An open-label monotherapy trial (NCT06453213) is ongoing, assessing safety and efficacy in adults with newly diagnosed or recurrent partial-onset seizures [92].

Preliminary real-world data show that CNB monotherapy may offer seizure control comparable to add-on therapy in suitable patients, with retention and tolerability profiles supportive of wider monotherapy use [93]. However, randomized controlled monotherapy trial data are still limited, and therefore, definitive conclusions await completion of ongoing studies [79].

**Tolerability and side effects of CNB monotherapy**

Regrettably, formal monotherapy safety data from randomized controlled trials are scarce; however, retrospective observational data suggest that CNB monotherapy is well tolerated, mirroring the safety profile observed in add-on therapy. Real-world evidence indicates that adverse events such as somnolence and dizziness are manageable, and serious adverse events remain infrequent [93]. Ongoing clinical trials aim to further clarify the safety and tolerability of CNB in monotherapy settings [92].

#### 3.3.2. Cenobamate as Add-On Therapy

CNB efficacy as an adjunctive treatment for drug-resistant focal epilepsy has been well-demonstrated in phase 2 and phase 3 clinical trials. In a pivotal phase 2 randomized, double-blind, placebo-controlled dose-response trial (NCT01866111), conducted between 2013 and 2015, 437 adults with uncontrolled focal seizures (despite treatment with 1–3 ASMs) were randomized to receive add-on CNB at doses of 100 mg, 200 mg, or 400 mg daily or placebo. The study showed significant dose-dependent reductions in median 28-day seizure frequency: 35.5% for 100 mg and 55.0% for both 200 and 400 mg groups, compared with 24.0% for placebo [76]. Responder rates (≥50% seizure reduction) during the maintenance phase were 40%, 56%, and 64%, respectively, versus 25% for placebo. Notably, seizure freedom rates were up to 21% with the highest dose, which is a very promising result in the treatment of this population [76,94]. These findings have been supported by an additional large phase 3 open-label safety study, further supporting CNB’s robust efficacy as an add-on therapy [94].

Real-world observational studies and meta-analyses have also confirmed the above-mentioned properties, showing sustained seizure reductions with acceptable tolerability in diverse clinical settings [77,78]. Importantly, analyses also suggest that earlier introduction of CNB (after prior failure of two antiseizure drugs) may improve patient outcomes, including higher seizure freedom and retention rates [95]. This still-evolving evidence supports CNB as an effective add-on therapeutic option for patients with drug-resistant focal epilepsy.

**Tolerability and side effects of CNB add-on therapy**

CNB (used as an adjunctive therapy) is generally well tolerated but associated with dose-dependent adverse effects. In the phase 2 pivotal trial, adverse events occurred in 65% to 90% of patients treated with CNB, versus 70% treated with placebo. Common side effects included dose-dependent somnolence, dizziness, fatigue, diplopia, and headache. Serious adverse events were reported in 4–9% of patients and included seizures and some neurological symptoms [76].

Early trials reported 3 cases of DRESS, all occurring during rapid dose escalation. This led to revised (slower and gradual) dosing protocols from low starting doses (12.5 mg daily). This modification successfully prevented further DRESS cases in larger, subsequent phase 3 open-label safety studies [94,96]. CNB has been also shown to cause dose-dependent QT interval shortening, which limits its use in patients with familial short QT syndrome or those on QT-shortening medications [28].

Due to CNB modulation of CYP enzymes, unfavorable significant drug–drug interactions can occur, requiring careful monitoring and adjustment of concomitant drugs (such as phenytoin, phenobarbital, clobazam, carbamazepine, and lamotrigine) [28]. Despite these complexities, CNB’s safety profile is comparable to or better than that of other add-on antiseizure drugs, supporting its broad clinical use in resistant focal epilepsy [96].

#### 3.3.3. Place of Cenobamate in Focal Epilepsy Therapy

CNB has emerged as a significant improvement in the treatment of focal-onset seizures, particularly in adults with drug-resistant epilepsy. Its dual and novel mechanism of action—targeting persistent sodium currents while also modulating GABA_A_ receptors—supports its superior efficacy and unique clinical profile compared to older ASMs [28,80]. Robust evidence from phase 2 and phase 3 clinical trials has proven CNB to have substantial efficacy as an add-on therapy, showing high rates of seizure frequency reductions and seizure freedom that exceed many currently available other treatment options [76,94]. CNB’s favorable pharmacokinetics and manageable safety profile (with the highlighted significance of careful dose titration) further enhance its clinical utility [28,78].

Although still primarily approved as adjunctive therapy, the expanding investigation into monotherapy and earlier introduction in the treatment protocol promise broader applicability and improved patient outcomes [92,93]. Real-world data confirm clinical trial findings, underscoring CNB’s enduring impact on seizure control and quality of life [78,93]. Current recommendations for CNB position it as a potent adjunctive option for patients inadequately managed with other antiseizure drugs, with the potential for future primary therapy roles [79,83]. Overall, CNB stands out as a promising and innovative therapeutic option, offering hope for patients with refractory focal epilepsy who have limited treatment alternatives.

## 4. Conclusions

Voltage-gated sodium channels remain one of the most important molecular targets in epilepsy therapy, and the development of newer-generation sodium channel modulators has significantly expanded therapeutic possibilities for patients with focal epilepsy. Unlike classical sodium channel blockers that predominantly affect fast inactivation and transient sodium currents, modern ASMs such as eslicarbazepine acetate, lacosamide, and cenobamate exhibit more selective and mechanistically diverse actions, including enhancement of slow inactivation and preferential inhibition of persistent sodium currents. These mechanisms allow for a more targeted suppression of pathological neuronal hyperexcitability while relatively sparing physiological neuronal signaling.

Eslicarbazepine acetate and lacosamide represent important advances within the group of sodium channel modulators due to their favorable pharmacokinetic properties, lower potential for drug–drug interactions, and generally good tolerability profiles. Both agents have demonstrated efficacy in monotherapy and adjunctive therapy across various patient populations, including elderly and pediatric patients. Their selective modulation of slow sodium channel inactivation appears particularly valuable in reducing excessive repetitive neuronal firing associated with focal seizures.

Among the discussed agents, cenobamate appears especially promising because of its unique dual mechanism involving preferential blockade of persistent sodium currents together with positive allosteric modulation of GABA_A_ receptors. Clinical trials and real-world studies consistently demonstrate high responder and seizure freedom rates, even in patients with drug-resistant epilepsy. These findings suggest that targeting persistent sodium currents may represent a major therapeutic advancement in modern epilepsy pharmacotherapy.

Overall, advances in sodium channel modulation have transformed the pharmacological management of epilepsy and continue to shape the development of more selective, effective, and better-tolerated ASMs. Eslicarbazepine acetate, lacosamide, and cenobamate exemplify the transition from conventional sodium channel blockade toward mechanism-driven therapies that may improve seizure control and quality of life in patients with epilepsy.

## Data Availability

No new data were created or analyzed in this study. Data sharing is not applicable.

## Abbreviations

The following abbreviations are used in this manuscript:

VGSC	Voltage-gated sodium channel
ASM	Antiseizure medication
GABA	Gamma-aminobutyric acid
INaT	Transient sodium current
INaP	Persistent sodium current
LCM	Lacosamide
ESL	Eslicarbazepine acetate
CNB	Cenobamate
OXC	Oxcarbazepine
VPA	Valproic acid
DRESS	Drug Reaction with Eosinophilia and Systemic Symptoms
BZD	Benzodiazepine
